# Risk factors for blood transfusion in adolescent patients with scoliosis undergoing scoliosis surgery: a study of 722 cases in a single center

**DOI:** 10.1186/s12891-020-03869-z

**Published:** 2021-01-05

**Authors:** Yulei Dong, Ning Tang, Shengru Wang, Jianguo Zhang, Hong Zhao

**Affiliations:** Department of Orthopedic Surgery, Peking Union Medical College Hospital, Chinese Academy of Medical Science and Peking Union Medical College, Beijing, 100730 China

**Keywords:** Scoliosis, Spine fusion, Blood transfusion, Osteotomy

## Abstract

**Background:**

To assess the risk factors for blood transfusion in a great number of adolescent cases with different types of scoliosis who received scoliosis surgery.

**Methods:**

Data of patients who were diagnosed as scoliosis and received one-stage posterior correction and spinal fusion from January 2014 to December 2017 were prospectively collected and retrospectively analyzed. Patients’ demographic characteristics, segments of spinal fusion, Cobb angle of the major curve,osteotomy pattern, preoperative and postoperative levels of hemoglobin, and allogeneic blood transfusion (ABT) were recorded and analyzed.

**Results:**

In this study, 722 cases with adolescent scoliosis were included, of whom 32.8% (237/722) received ABT. Risk factors included diagnosis: neurofibromatosis (OR = 5.592), syndromic (OR = 3.029),osteotomy: Ponte osteotomy (OR = 5.997), hemivertebrae resection (OR = 29.171), pedicle subtraction osteotomy (PSO)(OR = 8.712), vertebral column resection (VCR)(OR = 32.265);fusion segments (OR = 1.224) and intraoperative blood loss (OR = 1.004). In the subgroup analysis of cases with idiopathic scoliosis, Ponte osteotomy (OR = 6.086), length of segments of spinal fusion (OR = 1.293), and intraoperative blood loss (OR = 1.001) were found as risk factors for ABT. Results of receiver operating characteristic (ROC) curve analysis revealed that length of segments of spinal fusion equal to 11.5 vertebrae was the best cutoff value for cases with idiopathic scoliosis who did not receive osteotomy in both ABT group and non-ABT group. In the subgroup analysis of congenital scoliosis, Ponte osteotomy (OR = 5.087), hemivertebra resection (OR = 5.457), PSO (OR = 4.055), VCR (OR = 6.940), and intraoperative blood loss (OR = 1.004) were risk factors for ABT.

**Conclusions:**

Method of diagnosis, osteotomy pattern, segments of spinal fusion, and intraoperative blood loss were risk factors for ABT in cases with adolescent scoliosis. In cases with idiopathic scoliosis, Ponte osteotomy and segments of spinal fusion longer than 11.5 vertebrae were risk factors for ABT. In cases with congenital scoliosis, osteotomy pattern was the main risk factor for ABT.

**Level of evidence:**

Level III.

## Background

Scoliosis surgery is one of the most challenging surgeries in orthopedics. The surgery often requires extensive soft tissue exposure and bone resection that may cause a significant risk of blood transfusion [1]. Allogeneic blood transfusion (ABT) is associated with an increased risk of surgical site and systemic infection, transmission of infectious agents, acute hemolytic reactions, and transfusion-related immunomodulation [2]. Moreover, both autologous and allogeneic transfusions contribute to longer duration of hospitalization and, consequently, increased hospital costs [3]. Blood conservation techniques, such as tranexamic acid, intraoperative cell salvage, and controlled hypotension during operation reduce the risk of ABT [4]. However, long segments of spinal fusion and osteotomy still cause a large amount of blood loss and necessitate blood transfusion. Studies have reported predictive factors for blood transfusion in scoliosis surgery; however, the majority of cases had idiopathic scoliosis [5–7]. The association between etiology of scoliosis and blood transfusion was scarcely reported. Congenital scoliosis often requires osteotomy, causing more blood loss than idiopathic scoliosis. A systemic analysis of scoliosis surgery and blood transfusion with respect to blood conservation in modern surgery is highly essential for clinical decision making. The present study aimed to evaluate the risk factors for blood transfusion in a great number of cases with different types of scoliosis who received scoliosis surgery.

## Methods

### Patients

Patients who were diagnosed as scoliosis and received one-stage posterior correction and posterior spinal fusion from January 2014 to December 2017 were included in the current study. The patients’ age was 10–18 years old. Besides, cases diagnosed as degenerative scoliosis, and those who underwent revision surgery, had preoperative anemia or coagulation disorders were excluded. The patients’ demographic characteristics and clinical data, such as preoperative and postoperative hemoglobin, blood transfusion, segments of spinal fusion, osteotomy pattern, Cobb angle of the major curve and correction rate were collected and analyzed.

### Surgical procedures

All the surgeries were performed by senior surgeons in our medical center that is one of the most well-known spinal deformity centers in China. A midline posterior approach was applied and paravertebral muscles were detached by electrocautery. Pedicle screws were inserted by using free-hand technique. Ponte osteotomy, hemivertebra resection, pedicle subtraction osteotomy (PSO) or vertebral column resection (VCR) was performed for severe and rigid scoliosis according to surgeons’ experience. The osteotomy was performed by traditional osteotome. Bone wax was applied during surgery for bleeding from cancellous bone. Fusion was obtained by decortication with autologous spinal process or allograft cancellous bone. For all the patients, a drainage tube was placed under the fascia and removed 48–72 h after the surgery. Intraoperative blood loss included the amount of blood in suction container and surgical sponges.

### Blood transfusion strategy

Tranexamic acid was infused at a loading dose of 1 g before the surgery with a maintenance dose of 10 mg/kg/h until the wound closure. Ringer’s lactate solution and hydroxyethyl starch solution were used for hemodilution after general anesthesia and before the surgery. Controlled hypotension with a mean arterial pressure (MAP) of 80 mmHg was maintained during the surgery. A cell saver was utilized intraoperatively in all the patients. Bipolar electrocautery was applied in all the cases. Intraoperative blood gas analysis was applied for patients with large amount of blood loss. Blood test was carried out at each morning 3 days after the surgery and before being discharged from hospital. If the hemoglobin level was lower than 70 g/L, allogeneic red blood cells were infused. If the hemoglobin level was 70–100 g/L, allogeneic red blood cells were infused when a significant symptom of anemia was observed, such as lower blood pressure and increased heart rate. Blood transfusion did not include transfusion of fresh frozen plasma, platelets, cryoprecipitate, or albumin. All the patients received erythropoietin (EPO; 10,000 Units/day) and intravenous iron sucrose (100–200 mg/day) for 3–5 days postoperatively.

### Statistical analysis

Statistical analysis was performed using SPSS 16.0 software (IBM, Armonk, NY, USA). All continuous variables were expressed as a mean ± standard deviation. The independent t-test was employed to compare the differences between the ABT group and non-ABT group. Categorical variables were compared using the Pearson Chi-square test. Independent factors related to the transfusion were identified by binary logistic regression analysis. *P* < 0.05 was considered statistically significant.

## Results

**Fig. 1:**
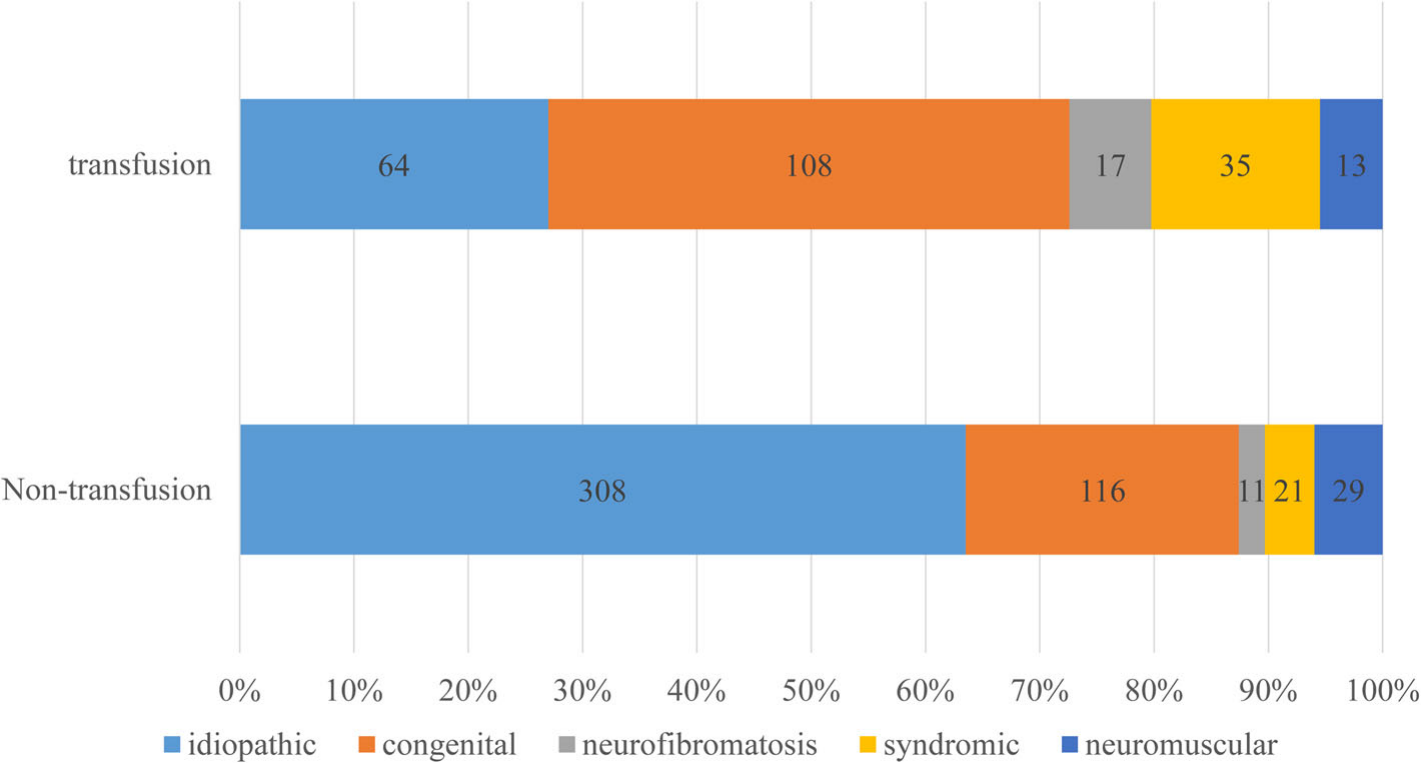
The relationship between blood transfusion rate and method of diagnosis. There were more cases with congenital scoliosis in ABT group compared to non-ABT group, while there was a greater proportion of cases with idiopathic scoliosis in non-ABT group than that in ABT group

**Fig. 2:**
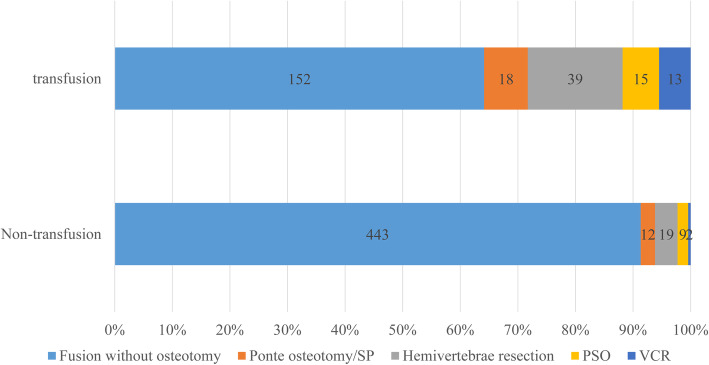
The relationship between blood transfusion rate and osteotomy pattern. There were a larger proportion of cases who received osteotomy in ABT group compared to non-ABT group

In the present study, 722 cases with adolescent scoliosis were included, of whom 32.8% (237/722) received ABT. The results of univariate analysis are shown in Table 1. The patients’ mean age in ABT group was slightly younger than that in non-ABT group, and there were more male patients in the ABT group. For classification of the etiology, the rate of diagnosis was different in the non-ABT group and ABT group. There were more cases with idiopathic scoliosis in non-ABT group compared to ABT group (63.5% vs. 27%), and more cases with congenital scoliosis in the transfusion group compared to non-ABT group (45.5% vs. 23.9%) (Fig. 1). The osteotomy pattern was also different in non-ABT group and ABT group. Additionally, 8.7% of cases in the non-ABT group underwent osteotomy, while 35.9% of cases in the ABT group received osteotomy (Fig. 2). The Cobb angle of the major curve was comparable between the two groups (53.5 ± 10.6 vs.57.5 ± 11.5). The length of segments of spinal fusion was markedly shorter in non-ABT group compared with that in ABT group (9.8 ± 2.7 vs. 11.6 ± 3.4, *P* = 0.000). The average blood loss and autologous blood transfusion were significantly decreased in the non-ABT group. The hemoglobin level was slightly lower at the postoperative day 1 (POD1) and POD3, while it became comparable at POD5.

**Table 1:** Univariate analysis between allogeneic blood transfusion group and non-transfusion group

	Non-Transfusion group	Transfusion group	Transfusion rate	*P*
Age (year)	14.4 ± 1.8	14.0 ± 2.0		0.013
BMI (kg/m2))	19.0 ± 3.4	18.5 ± 3.7		0.205
Gender, male/total (%)	128/485 (26.4%)	85/237 (35.9%)		0.009
Diagnosis, n(%)	485 (100%)	237 (100%)		0.000
Idiopathic, n(%)	308 (63.5%)	64 (27%)	17.2%	
Congenital, n(%)	116 (23.9%)	108 (45.5%)	48.2%	
Neurofibromatosis, n(%)	11 (2.3%)	17 (7.2%)	60.7%	
Syndromic, n(%)	21 (4.3%)	35 (14.8%)	62.5%	
Neuromuscular, n(%)	29 (6.0%)	13 (5.5%)	31.0%	
Osteotomy pattern, n(%)	485 (100%)	237 (100%)		0.000
Fusion without osteotomy, n(%)	443 (91.3%)	152 (64.1%)	25.5%	
Ponte osteotomy, n(%)	12 (2.5%)	18 (7.6%)	60%	
Hemivertebrae resection, n(%)	19 (3.9%)	39 (16.5%)	67.2%	
PSO, n(%)	9 (1.9%)	15 (6.3%)	62.5%	
VCR, n(%)	2 (0.4%)	13 (5.5%)	86.7%	
Cobb angle of major curve-preoperative, degree	53.5 ± 10.6	57.5 ± 11.5		0.108
Cobb angle of major curve-postoperative, degree	18.5 ± 9.0	16.5 ± 8.5		0.309
Major curve correction rate,%	62 ± 7.6	59.5 ± 6.4		0.205
Fusion segments	9.8 ± 2.7	11.6 ± 3.4		0.000
Preoperative HGB, g/L	136.2 ± 11.7	135.3 ± 14.0		0.356
Intraoperative Blood loss, mL	414.5 ± 235.6	839.4 ± 573.7		0.000
Intraoperative cell salvage, mL	200.9 ± 166.8	397.6 ± 323.7		0.000
HGB POD1, g/L	108.2 ± 15.0	111.1 ± 16.0		0.018
HGB POD3, g/L	102.7 ± 14.1	105.4 ± 14.9		0.041
HGB POD5, g/L	103.2 ± 13.9	105.8 ± 15.2		0.273

The results of analysis of risk factors are summarized in Table 2. The diagnosis, methods of osteotomy, segments of spinal fusion, and intraoperative blood loss were the most significant risk factors. Patients with neurofibromatosis scoliosis (odds ratio (OR) = 5.592, *P* = 0.001) and syndromic scoliosis (OR = 3.029, *P* = 0.004) had a significantly higher incidence of ABT. Ponte osteotomy (OR = 5.997, *P* = 0.000), hemivertebra resection (OR = 29.171, *P* = 0.000), PSO (OR = 8.712, *P* = 0.018), and VCR (OR = 32.265, *P* = 0.002) showed a significantly higher incidence of ABT in patients who received osteotomy compared with those who did not. Segments of spinal fusion (OR = 1.224, *P* = 0.000) and intraoperative blood loss (OR = 1.004, *P* = 0.000) were also found as independent risk factors for ABT.

**Table 2:** Multivariate analysis of independent risk factors associated with allogeneic blood transfusion

factors	Odds ratio	95%CI	*P*
**Diagnosis**			0.001
Idiopathic	1	1	
Congenital	1.716	0.985–2.991	0.057
Neurofibromatosis	5.592	2.065–15.144	0.001
Syndromic	3.029	1.434–6.396	0.004
Neuromuscular	1.048	0.418–2.629	0.920
**Osteotomy**			0.000
Fusion without osteotomy	1	1	
Ponte osteotomy	5.997	2.266–15.872	0.000
Hemivertebrae resection	29.171	9.491–89.658	0.000
PSO	8.712	1.449–52.368	0.018
VCR	32.265	3.673–283.462	0.002
Gender			
Male	1	1	
Female	1.474	0.873–2.489	0.147
Age	0.881	0.781–0.994	0.040
Fusion segments	1.224	1.115–1.345	0.000
Intraoperative Blood loss	1.004	1.002–1.005	0.000
Intraoperative cell salvage	0.998	0.997–1.000	0.097

**Fig. 3:**
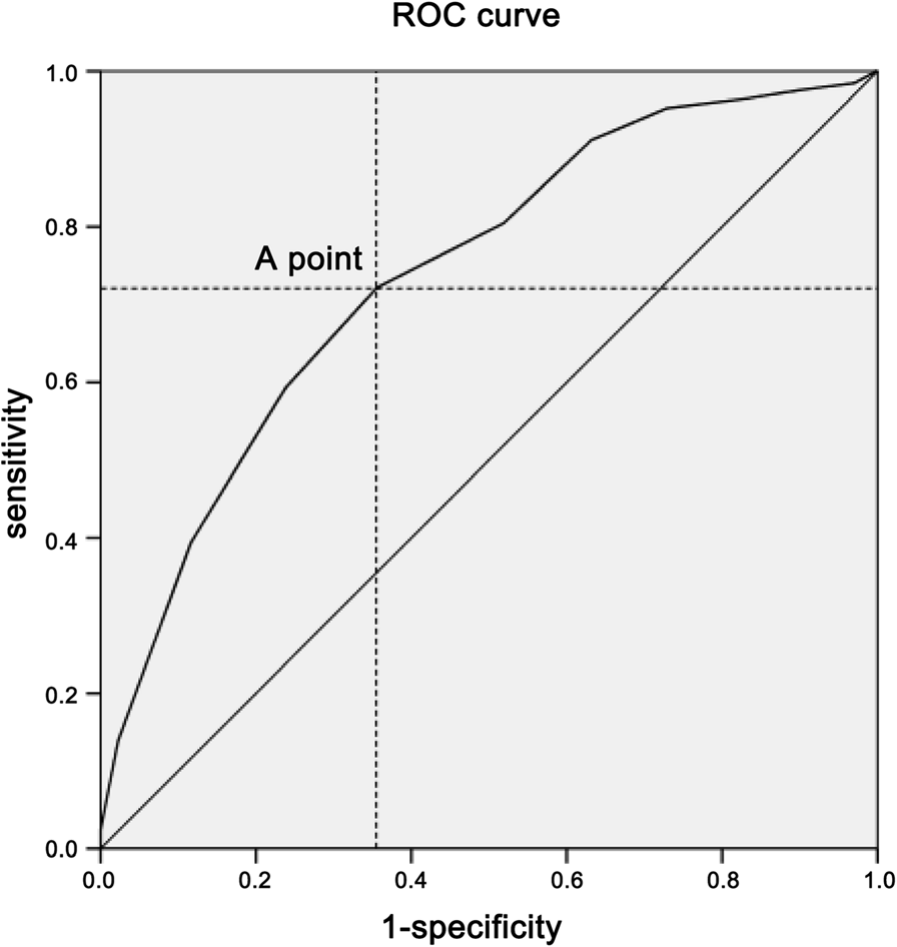
Subgroup analysis was carried out in patients with idiopathic scoliosis who received fusion surgery without osteotomy. ROC curve analysis revealed that in fused segments, 11.5 vertebrae was the best cutoff value in ABT group and non-ABT group (sensitivity, 72.1%; specificity, 64.5%)

The results of subgroup analysis of cases with idiopathic scoliosis are shown in Table 3. The transfusion rate in cases with idiopathic scoliosis was 17.2% (64/372). In addition, the proportion of cases that underwent Ponte osteotomy was notably higher in ABT group compared with that in non-ABT group (10.9% vs. 1.3%, *P* = 0.000). The major curve correction rate was a little higher in the ABT group (72.0 ± 9.5 vs.74.5 ± 8.4), however, it didn’t reach statistical significance. The length of segments of spinal fusion was significantly shorter in non-ABT group than that in ABT group (9.6 ± 2.7 vs. 12.1 ± 2.6, *P* = 0.000). Results of multivariate regression analysis indicated that Ponte osteotomy (OR = 6.086, *P* = 0.000) and segments of spinal fusion (OR = 1.293, *P* = 0.000) were independent risk factors for ABT. Receiver operating characteristic (ROC) curve analysis revealed that 11.5 was the best cutoff segment for idiopathic scoliosis cases without undergoing osteotomy in ABT and non-ABT groups (Fig. 3; sensitivity, 72.1%; specificity, 64.5%) (Table 4).

**Table 3:** Univariate analysis between allogeneic blood transfusion group and non-transfusion group in idiopathic scoliosis

	Non-transfusion group	Transfusion group	*P*
Age (year)	14.7 ± 1.7	14.7 ± 1.8	0.803
BMI (kg/m2))	18.8 ± 3.1	18.9 ± 3.9	0.928
Gender, male/total (%)	54/308	15/64	0.269
Osteotomy pattern, n(%)	308 (100%)	64 (100%)	0.000
Fusion without osteotomy, n(%)	304 (98.7%)	57 (89.1%)	
Ponte osteotomy, n(%)	4 (1.3%)	7 (10.9%)	
Cobb angle of major curve-preoperative, degree	57.5 ± 11.5	59.5 ± 12.5	0.068
Cobb angle of major curve-postoperative, degree	13.5 ± 6.0	12.5 ± 5.5	0.189
Major curve correction rate, %	72.0 ± 9.5	74.5 ± 8.4	0.091
Fusion segments	9.6 ± 2.7	12.1 ± 2.6	0.000
Preoperative HGB, g/L	134.9 ± 11.7	133.3 ± 17.1	0.366
Intraoperative Blood loss, mL	400.2 ± 243.9	713.6 ± 394.1	0.000
Intraoperative cell salvage, mL	190.7 ± 165.8	358.6 ± 246.6	0.000
HGB POD1, g/L	107.6 ± 14.4	109.2 ± 19.3	0.452
HGB POD3, g/L	102.3 ± 13.7	104.6 ± 15.2	0.335
HGB POD5, g/L	102.4 ± 15.1	107.5 ± 11.8	0.268

**Table 4:** Multivariate analysis of independent risk factors associated with allogeneic blood transfusion in idiopathic scoliosis

Factors	Odds ratio	95%CI	*P*
**Osteotomy**			0.000
Fusion without osteotomy	1	1	
Ponte osteotomy	6.086	3.894–9.513	0.000
Fusion segments	1.293	1.232–1.357	0.000
Intraoperative Blood loss	1.001	1.001–1.002	0.000
Intraoperative cell salvage	1.000	1.000–1.001	0.188

The results of subgroup analysis of congenital scoliosis are presented in Table 5. The transfusion rate of congenital scoliosis was 48.2% (108/224). The proportion of cases that received osteotomy was remarkably higher in ABT group compared with that in non-ABT group (62% vs. 29.3%, *P* = 0.000). The segments of spinal fusion and were comparable between the two groups (ABT group (10.3 ± 2.7) vs. non-ABT group (10.6 ± 4.1, *P* = 0.518)). The Cobb angle of major curve and the correction rate were also comparable in the two groups. Multivariate regression analysis results of independent risk factors of congenital scoliosis are summarized in Table 6. Ponte osteotomy (OR = 5.087, *P* = 0.025), hemivertebra resection (OR = 5.457, *P* = 0.000), PSO (OR = 4.055, *P* = 0.015), and VCR (OR = 6.940, *P* = 0.024) were all associated with high risk of blood transfusion.

**Table 5:** Univariate analysis between allogeneic blood transfusion group and non-transfusion group in congenital scoliosis

	Non-transfusion group	Transfusion group	*P*
Age (year)	13.7 ± 1.8	13.7 ± 2.0	0.894
BMI (kg/m2))	19.2 ± 3.7	19.1 ± 3.9	0.865
Gender, male/total (%)	46/116 (39.7%)	43/108 (39.8%)	0.981
Osteotomy pattern, n(%)	116 (100%)	108 (100%)	0.000
Fusion without osteotomy, n(%)	82 (70.7%)	41 (38.0%)	
Ponte osteotomy, n(%)	4 (3.4%)	6 (5.6%)	
Hemivertebrae resection, n(%)	19 (16.4%)	39 (36.1%)	
PSO, n(%)	9 (7.8%)	13 (12.0%)	
VCR, n(%)	2 (1.7%)	9 (8.3%)	
Cobb angle of major curve-preoperative, degree	60.5 ± 9.5	61.3 ± 8.8	0.338
Cobb angle of major curve-postoperative, degree	23.5 ± 7.5	25.5 ± 8.4	0.209
Major curve correction rate,%	58.4 ± 10.5	59.3 ± 9.4	0.101
Fusion segments	10.3 ± 2.7	10.6 ± 4.1	0.518
Preoperative HGB, g/L	137.7 ± 10.3	136.6 ± 12.6	0.444
Intraoperative Blood loss, mL	425.8 ± 229.4	843.7 ± 678.9	0.000
Intraoperative cell salvage, mL	204.7 ± 168.6	398.7 ± 361.9	0.000
HGB POD1, g/L	109.3 ± 15.4	113.6 ± 15.1	0.039
HGB POD3, g/L	102.5 ± 15.2	108.4 ± 14.9	0.016
HGB POD5, g/L	103.1 ± 13.3	109.7 ± 17.0	0.158

**Table 6:** Multivariate analysis of independent risk factors associated with allogeneic blood transfusion in congenital scoliosis

factors	Odds ratio	95%CI	*P*
**Osteotomy**			0.000
Fusion without osteotomy	1	1	
Ponte osteotomy	5.087	1.224–21.139	0.025
Hemivertebrae resection	5.457	2.462–12.095	0.000
PSO	4.055	1.314–12.517	0.015
VCR	6.940	1.284–37.496	0.024
Intraoperative blood loss	1.004	1.002–1.006	0.000
Intraoperative cell salvage	1.000	0.997–1.003	0.977

## Discussion

Scoliosis surgery aims to reduce the abnormal curve in the spine and prevent it from getting worse. Several studies have reported the risk factors for massive blood transfusion in patients with adolescent scoliosis [1, 4–8]. The results of the current study showed that 32.8% (237/722) of patients with different types of scoliosis received ABT. Thus, it is of great significance to be aware of the characteristics of blood transfusion preoperatively for clinical decision making.

According to the literature, the transfusion rate in cases with idiopathic scoliosis ranged from 1.7 to 67.6% [8, 9]. Hassan et al. studied 110 patients with scoliosis, and it was demonstrated that the transfusion rate was only 1.7% (1/60) for cases with idiopathic scoliosis. In that research, patients received preoperative evaluation, cell salvage, topical hemostasis, antifibrinolytics, and hypotensive anesthesia. Their results indicated that implementation of a blood management protocol resulted in a low transfusion rate and unexpectedly led to the preoperative diagnosis in a number of patients with a low level of von Willebrand factor activity [9]. In a recently conducted research, ABT was noted in 73 (18.2%) out of 402 patients with idiopathic scoliosis [7], which is quite similar to the findings of the present study. In our research, the blood transfusion rate in cases with idiopathic scoliosis was 17.2% (64/372). Our findings were almost the same except for two surgical strategies, which might lead to a faster surgery, reduced intraoperative blood loss, and a reduced risk of ABT [10, 11].

Longer segments of spinal fusion were noted to be associated with a higher risk of ABT. In the present study, we found that segments of spinal fusion longer than 11.5 vertebrae had the best cutoff value in ABT group and non-ABT group. A similar conclusion was drawn from a National Surgical Quality Improvement Program pediatric database from 2012 to 2013. They concluded that posterior arthrodesis of 13 or more vertebral segments (*P* < 0.001) were independent risk factors for requiring blood transfusion in AIS patients [8]. This conclusion was quite useful before surgery which implies selective thoracic or lumbar fusion have very low incidence of blood transfusion.

Congenital scoliosis is a failure of vertebral formation and/or segmentation arising from abnormal vertebral development during gestation. Neuromuscular scoliosis (NMS) refers to a non-congenital spinal deformity that occurs in patients with any type of pre-existing neuromuscular diagnosis. NMS may affect patients of any age, and progress relentlessly in many cases, particularly in patients with more severe neurologic and systemic involvement. Hassan et al. studied 28 cases with NMS, of whom 36% required ABT, which is quite similar to the rate achieved in the current research (13/42, 31%) [9]. Claire et al. investigated 147 patients with scoliosis, and found that incidence of congenital scoliosis and NMS was 21.4 and 76.5%, respectively. Their results indicated that the blood transfusion rate was only 9.4% in patients with idiopathic scoliosis, which was notably lower than that in cases with congenital scoliosis and NMS [12]. Yu et al. assessed predictors of massive blood loss after scoliosis surgery, and it was concluded that the risk of massive blood loss in patients with scoliosis could increase if they had preoperative Cobb angle > 50° or aimed to undergo osteotomy or fusion of more than 6 levels [13]. To our knowledge, the number of levels to be fused is a good predictor of massive blood loss in scoliosis surgery. In our research, the Cobb angle of the major curve was comparable between ABT and non-ABT group. We defined the osteotomy pattern for each case, and it seems to be more important than Cobb angle. The analysis of independent risk factors showed that diagnosis of neurofibromatosis was associated with a high OR of 5.592 compared to idiopathic scoliosis. This could be explained by malformation of pedicle in neurofibromatosis scoliosis, which may cause failure in pedicle screw fixation, thereby increasing the exposure time [14, 15].

Osteotomy often causes a large amount of blood loss. In the present research, the transfusion rate in cases who underwent osteotomy varied between 60 and 86.7%. For congenital scoliosis, the main risk factor for blood transfusion is osteotomy with an OR value greater than 4. Chang et al. estimated that the blood loss of VCR was 1916 ml [16]. To reduce the incidence of transfusion in cases with a high level of osteotomy, further effective approaches are required, and blood conservation protocols remain to be updated.

The advantage of our study is the large case sample which is quite essential to determine the true value of transfusion rate. All patients received consistent blood saving scheme. Subgroup analysis was carried out between different diagnosis and surgical methods, which is useful in surgery decision making process. However, there are several shortcomings in the current study. Firstly, duration of surgery were not measured for each case separately. Secondly, its retrospective nature is one of the main limitations.

## Conclusions

In summary, method of diagnosis, osteotomy pattern, segments of spinal fusion, and intraoperative blood loss were found as risk factors for blood transfusion in cases with adolescent scoliosis. In cases with idiopathic scoliosis, Ponte osteotomy and segments of spinal fusion longer than 11.5 vertebrae were risk factors for ABT. In cases with congenital scoliosis, osteotomy pattern was the main risk factor for ABT.

## Data Availability

Please contact the corresponding author for scientific use.

## Abbreviations

PSO: Pedicle subtraction osteotomy
MAP: Mean arterial pressure
POD: Postoperative day
VCR: Vertebral column resection
